# Exercise-Induced Hypoxaemia Developed at Sea-Level Influences Responses to Exercise at Moderate Altitude

**DOI:** 10.1371/journal.pone.0161819

**Published:** 2016-09-01

**Authors:** Anne-Fleur Gaston, Fabienne Durand, Emma Roca, Grégory Doucende, Ilona Hapkova, Enric Subirats

**Affiliations:** 1 Laboratoire Européen Performance Santé Altitude—EA4604, Université de Perpignan Via Domitia, Font-Romeu, France; 2 Facultat de Medicina, Universitat de Girona, Girona, Spain; Université Claude Bernard Lyon 1, FRANCE

## Abstract

**Purpose:**

The aim of this study was to investigate the impact of exercise-induced hypoxaemia (EIH) developed at sea-level on exercise responses at moderate acute altitude.

**Methods:**

Twenty three subjects divided in three groups of individuals: highly trained with EIH (n = 7); highly trained without EIH (n = 8) and untrained participants (n = 8) performed two maximal incremental tests at sea-level and at 2,150 m. Haemoglobin O_2_ saturation (SpO_2_), heart rate, oxygen uptake (VO_2_) and several ventilatory parameters were measured continuously during the tests.

**Results:**

EIH athletes had a drop in SpO_2_ from 99 ± 0.8% to 91 ± 1.2% from rest to maximal exercise at sea-level, while the other groups did not exhibit a similar decrease. EIH athletes had a greater decrease in VO_2max_ at altitude compared to non-EIH and untrained groups (-22 ± 7.9%, -16 ± 5.3% and -13 ± 9.4%, respectively). At altitude, non-EIH athletes had a similar drop in SpO_2_ as EIH athletes (13 ± 0.8%) but greater than untrained participants (6 ± 1.0%). EIH athletes showed greater decrease in maximal heart rate than non-EIH athletes at altitude (8 ± 3.3 bpm and 5 ± 2.9 bpm, respectively).

**Conclusion:**

EIH athletes demonstrated specific cardiorespiratory response to exercise at moderate altitude compared to non-EIH athletes with a higher decrease in VO_2max_ certainly due to the lower ventilator and HR_max_ responses. Thus EIH phenomenon developed at sea-level negatively impact performance and cardiorespiratory responses at acute moderate altitude despite no potentiated O_2_ desaturation.

## Introduction

In endurance sport, most training camps and competitions take place at altitude around 2000 m. At this altitude, aerobic performance and maximal oxygen uptake (VO_2max_) are reduced due to the decrease in partial pressure of inspired O_2_ (PIO_2_) [1]. However, altitude does not appear to affect every individual in an equal manner. Indeed it is now well documented that endurance-trained athletes demonstrate a larger decline in VO_2max_ with increasing altitude compared to untrained subjects [2–4]. This particular sensitivity of endurance-trained athletes to moderate altitude has been related to an important decrease in arterial O_2_ saturation (SaO_2_) [2,3,5–7]. At sea level, 50% of healthy endurance-trained athletes develop exercise-induced hypoxaemia (EIH) [8]. “EIH athletes” show a difference between rest and maximal arterial O_2_ pressure (PaO_2_) values for at least 10 mmHg and/or a delta of SaO_2_ of at least 4% [9]. It is reported that EIH subsequently affects performance in response to high intensity exercise [10]. Physiopathology of EIH seems to be 1) a relative hypoventilation related to training adaptation and 2) gas exchange abnormality [11]. Each of these mechanisms appear to participate in EIH occurrence, with specific contribution depending on the training status and/or exercise mode and/or muscle mass involved [12]. However on average ~20% of the variation in PaO_2_ between individuals during exercise is due to the variation in hyperventilation response, with the remaining ~80% of the variance in PaO_2_ drop roughly divided evenly between ventilation-perfusion mismatch and diffusion limitation [13]. Because PIO_2_ is reduced with altitude, EIH exhibited at sea level by some endurance-trained athletes could be amplified at acute moderate altitude. Only a few studies have examined the consequences of EIH during a maximal exercise performed at altitude while the number of athletes involved in endurance mountaineering sports, who are prone to develop EIH, was greatly increased in the last decade. In fact, literature reports only four studies that have compared O_2_ desaturation and aerobic performance during maximal exercise at altitude between EIH athletes identified at sea level and non-EIH athletes [4,6,14,15]. If these studies have reported a greater decrease in VO_2max_ in EIH athletes than in non-EIH athlete, they also have reported controversial results about SpO_2_ decrement and cardio-respiratory responses. Moreover none of these studies has examined the effect of a moderate altitude despite it is an usual altitude for training camps and competitions in endurance sports or mountaineering. The general aim of this study was to better understand the constraints applied on the cardiorespiratory system of EIH athletes at moderate altitude. More specifically, the purpose of this study was to evaluate 1) the level of O_2_ desaturation and 2) cardio-ventilatory responses of EIH athletes identified at sea level during a maximal exercise performed at acute moderate altitude.

## Materials and Methods

### Participants

Twenty-three healthy non-smoker males were recruited for this study. All the participants were sea-level residents. Fifteen participants were endurance-trained athletes involved in running or cycling activities (at least 10 hours per week for the past ten years). The remaining eight were sedentary or active in recreational sports, but had never been engaged in systematic endurance training. Before their inclusion in the protocol, each participant was informed about the procedures and the potential risks inherent to the experiments. They signed a written informed consent form. All procedures were approved by the ethics committee of the Consell General de l’Esport, Catalunya.

### Design

Each participant was involved in two maximal incremental tests performed on a cycle ergometer (Kettler, Ense-Parsit, Germany). The exercises were separated by 7 days. The first test was performed at sea level (SL) and the second at 2150 meters in an altitude hut (ALT). After a 6-minutes warm-up at 30 Watts (W) for untrained participants and 60 W for trained participants, the power was increased every minute by 30 W until exhaustion. The test was considered to be maximal if at least three of the following criteria were presented: 1) an increase of VO_2_ of < 100 ml with the last increase in work rate, 2) achievement of age-predicted maximal heart rate (HR) [210-(0.65 age) ± 10%], 3) a respiratory exchange ratio (RER) above 1.1, and 4) the incapacity to maintain the pedalling frequency imposed (70 rpm minimum) despite maximum effort and verbal encouragement. During each test, cardiorespiratory parameters and O_2_ saturation were recorded. SL test allowed us to determine two groups of endurance-trained athletes: one group exhibiting EIH (n = 7) and a second group without EIH (n = 8). Mean characteristics of the three groups are presented in Table 1.

**Table 1:** Anthropometric and training data.

	EIH	Non-EIH	Untrained
Age (years)	40 ± 3.5	39 ±	40 ± 1.6
Body mass (kg)	70 ± 7.2 *	69 ± 7.3 *	77 ± 5.9
Height (cm)	177 ± 5.2	175 ± 6.2	173 ± 3.0
Body mass index (kg.m^-2^)	22 ± 2.2 *	22 ± 1.3 *	25 ± 1.7
Training (hours.week^-1^)	14 ± 5.7 *	11 ± 4.7 *	2 ± 1.3
Training (years)	22 ± 5.7	21 ± 6.3	/

* Significantly different from untrained group (p < 0.05).

### Measurements and EIH definition

Oxygen saturation level of haemoglobin was assessed by the peripheral capillary oxygen saturation (SpO_2_). SpO_2_ was measured continuously during tests using an ear-lobe pulse oximeter (Nonin, Minnesota, USA). PureSAT® technology used in Nonin Medical pulse oximeter guarantees an measurement accuracy of ± 2.1% compared to the gold standard which is CO-oximetry analysis of arterial blood samples. The ear was pre-warmed by a vasodilating capsaicin cream (Finalgon, Fher, Spain) to avoid poor perfusion during exercise. EIH may be considered to exist when SpO_2_ decreases of 4% between rest and maximal effort of the test at SL of at least the last 3 minutes [9]. At the same time, respiratory data were collected by a portable automatic breath-by-breath metabolic system (K4b^2^, Cosmed, Rome, Italy): VO_2_ (ml.min^-1^.kg^-1^), RER (VCO_2_/VO_2_), minute ventilation (VE, l.min^-1^), tidal volume (V_T_, l), breathing frequency (B_F_, breaths.min^-1^). K4b^2^ system was calibrated before each test according to the manufacturer’s specifications: using a 3-l syringe and a gas bottle of known O_2_ and CO_2_ concentrations (16 and 5%, respectively). Each subject was also equipped with a chest belt (Polar Electro, Kempele, Finland) to collect HR continuously (beats.min^-1^).

### Statistical treatment

The results are expressed as means **±** SD. Differences with no repeated measures among the three groups were analysed using one-way analysis of variance (ANOVA). Two-way ANOVA were used for repeated measures to analyse the main effect and interaction of altitude and group on measured parameters. Correlations between the variables were tested using Pearson’s product-moment correlation coefficient test. For all tests, the level of statistical significance was set at p < 0.05. Analyses were conducted using SigmaStat software (Ver 3.5).

## Results

### Characteristics at rest and in response to exercise at SL

**Fig 1:**
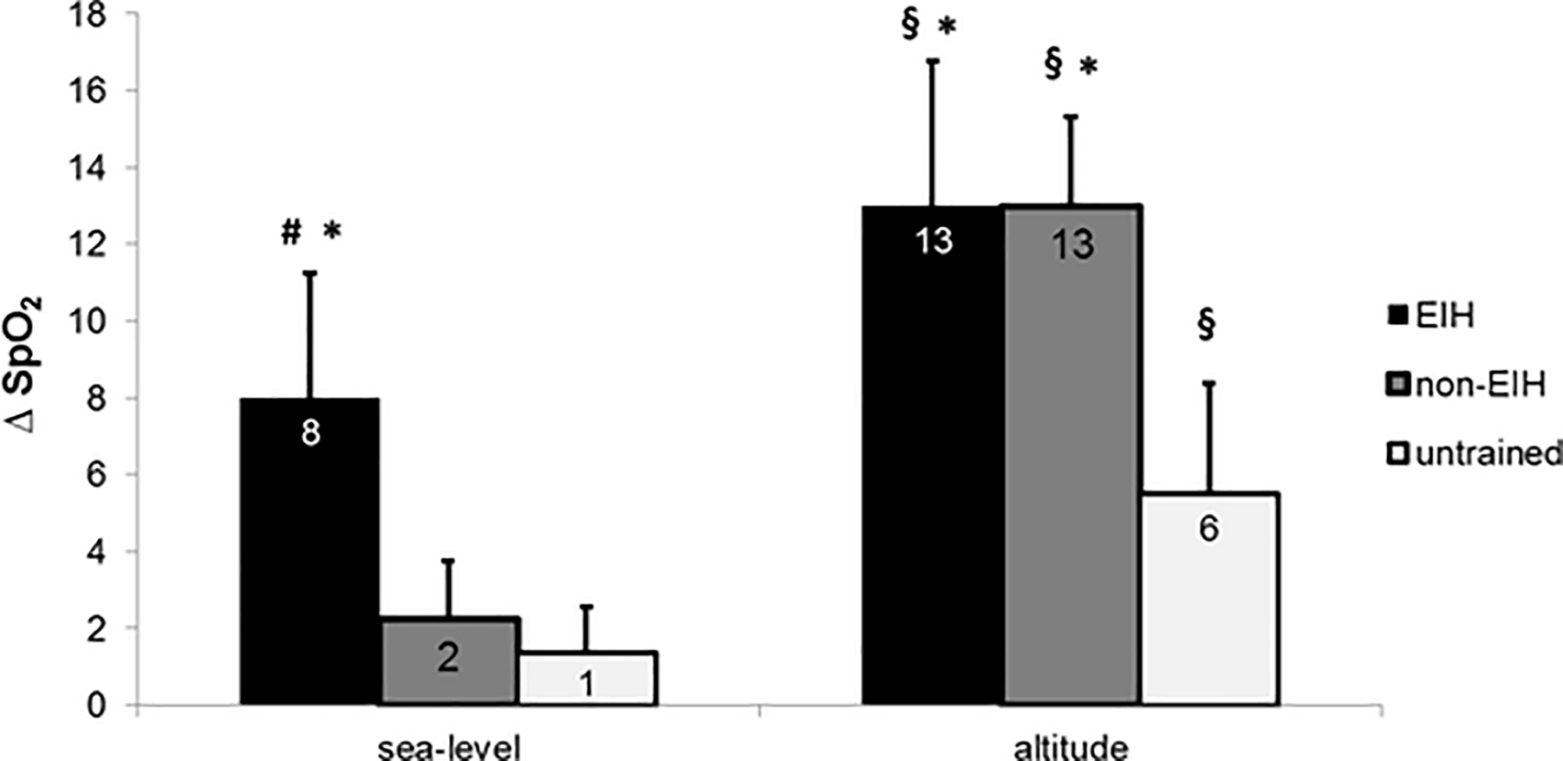
Delta of haemoglobin O_2_ saturation (ΔSpO_2_, difference between rest and maximal exercise values) at sea level and at 2150 meters for EIH, non-EIH and untrained participants. § Significantly different from sea level (p < 0.01); * Significantly different from untrained participants (p < 0.01); # Significantly different from non-EIH (p < 0.01).

**Fig 2:**
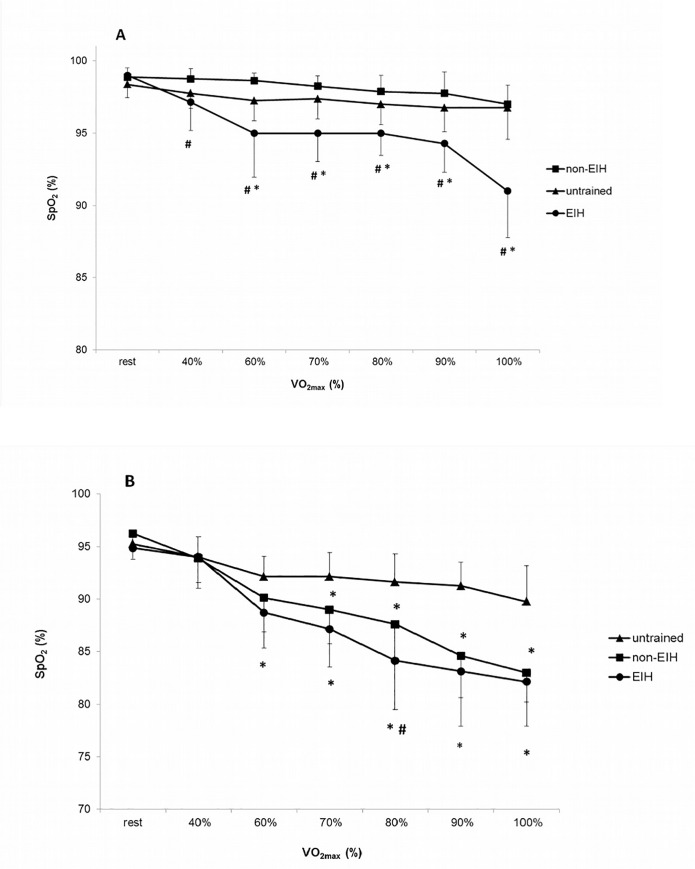
Kinetics of haemoglobin O_2_ saturation (SpO_2_) during rest and incremental maximal exercise at sea level (A) and altitude (B). * Significantly different from untrained (p < 0.05); # Significantly different from non-EIH (p < 0.05). Note that, at all intensities, SpO_2_ values at altitude are significantly different from sea level in the three groups (p < 0.05).

There was no difference in anthropometric data between EIH and non-EIH groups (Table 1). Untrained group had a higher body mass and body mass index compared to athletes (p < 0.05). Untrained participants presented significant differences in physiological data compared to endurance-trained groups (Table 1). At SL, there was no significant difference in SpO_2_ resting values between groups. SpO_2_ was normal (99 ± 0.8%) for all the participants at rest. Seven of the 15 endurance-trained athletes (~50%) exhibited EIH at SL. Delta of SpO_2_ (ΔSpO_2,_ difference between rest and end of exercise values) at SL was significantly higher in EIH group than in non-EIH and untrained groups (Fig 1). Regarding SpO_2_ kinetics at SL in EIH group reported a greater SpO_2_ drop from the beginning until 60% of VO_2max_ and the difference was reinforced from 90% to 100% of VO_2max_ (Fig 2A). Performance and cardiorespiratory maximal parameters are presented in Table 2. Endurance-trained groups had a significant greater VO_2max_ and P_max_ than untrained group. EIH group exhibited a lower ventilatory equivalent for O_2_ (VE/VO_2max_) and CO_2_ (VE/VCO_2max_) at maximal exercise while no difference occurred in HR_max_, B_Fmax_, V_Tmax_, VE_max_ and duration of test.

**Table 2:** Incremental maximal exercise parameters at sea level and at altitude.

		EIH	Non-EIH	Untrained
Effective		7	8	8
**VO**_**2max**_**(**ml.min^-1^.kg^-1^)	SL	62 ± 2.9 *	59 ± 2.9 *	41 ± 5.1
	ALT	48 ± 5.4 *§	51 ± 4.1 *§	36 ± 7.9 §
**P-VO**_**2max**_ **(**Watt)	SL	424 ± 53.2 *	383 ± 47.4 *	315 ± 39.3
	ALT	373 ± 75.2 *§	341 ± 45.2 *§	285 ± 45.4 §
**HR**_**max**_ (beats.min^-1^)	SL	176 ± 8.5	173 ± 7.3	180 ± 8.3
	ALT	168 ± 9.2 §	168 ± 9.1	176 ± 8.0
**VE**_**max**_ (l.min^-1^)	SL	149 ± 27.4	163 ± 27.8 *	135 ± 22.1
	ALT	136 ± 44.1	152 ± 23.9	136 ± 28.8
**B**_**Fmax**_ (breaths.min^-1^)	SL	46 ± 10.5	54 ± 7.0	48 ± 8.5
	ALT	40 ± 10.0 #	49 ± 9.7	48 ± 8.6
**V**_**Tmax**_ (l)	SL	3.3 ± 0.5	3.0 ± 0.5	2.8 ± 0.3
	ALT	3.3 ± 0.5	3.2 ± 0.5	2.8 ± 0.4
**VE/VO**_**2max**_	SL	35 ± 4.7 *#	40 ± 3.9	43 ± 6.9
	ALT	40 ± 7.2 *	44 ± 7.5	49 ± 4.3 §
**VE/VCO**_**2max**_	SL	30 ± 3.8 #	34 ± 2.5	34 ± 4.4
	ALT	32 ± 5.1 *#	36 ± 3.9	37 ± 2.8

VO_2max_: maximal oxygen uptake; P-VO_2max_: power achieved at VO_2max_; HR_max_: heart rate at maximal exercise; VE_max_: minute ventilation at maximal exercise; B_Fmax_: breathing frequency at maximal exercise; V_Tmax_: tidal volume at maximal exercise; VE/VO_2max_: ventilatory equivalent for O_2_ at maximal exercise; VE/VCO_2max_: ventilatory equivalent for CO_2_ at maximal exercise.

§ Significantly different from sea level (p < 0.05);

* Significantly different from untrained participants (p < 0.05);

# Significantly different from non-EIH athletes (p < 0.05).

### Adaptations during exercise at ALT

At 2150 m, PIO_2_ is decreased and there was a significant effect of hypoxia on SpO_2_ resting values (99 ± 0.8% vs 95 ± 1.3% over all subjects) without difference among groups. SpO_2_ values at the end of exercise were significantly reduced in the three groups compared to SL condition, respectively: 82 ± 4.2% vs 91 ± 1.2% in EIH group; 83 ± 2.8% vs 97 ± 1.3% in non-EIH group; 90 ± 3.5% vs 97 ± 1.8% in untrained group (Fig 2). ΔSpO_2_ was significantly greater in EIH and non EIH groups compared with untrained group (Fig 1). Non-EIH group defined at SL have developed the same O_2_ desaturation than EIH group defined at SL. In all groups, there was an effect of ALT condition on ΔSpO_2,_ reflecting O_2_ desaturation during exercise. Regarding SpO_2_ kinetic, most of SpO_2_ decrease occurred between 60 to 80% of VO_2max_ in EIH group whereas it decrease between 80 to 100% of VO_2max_ in non-EIH trained group (Fig 2B).

**Fig 3:**
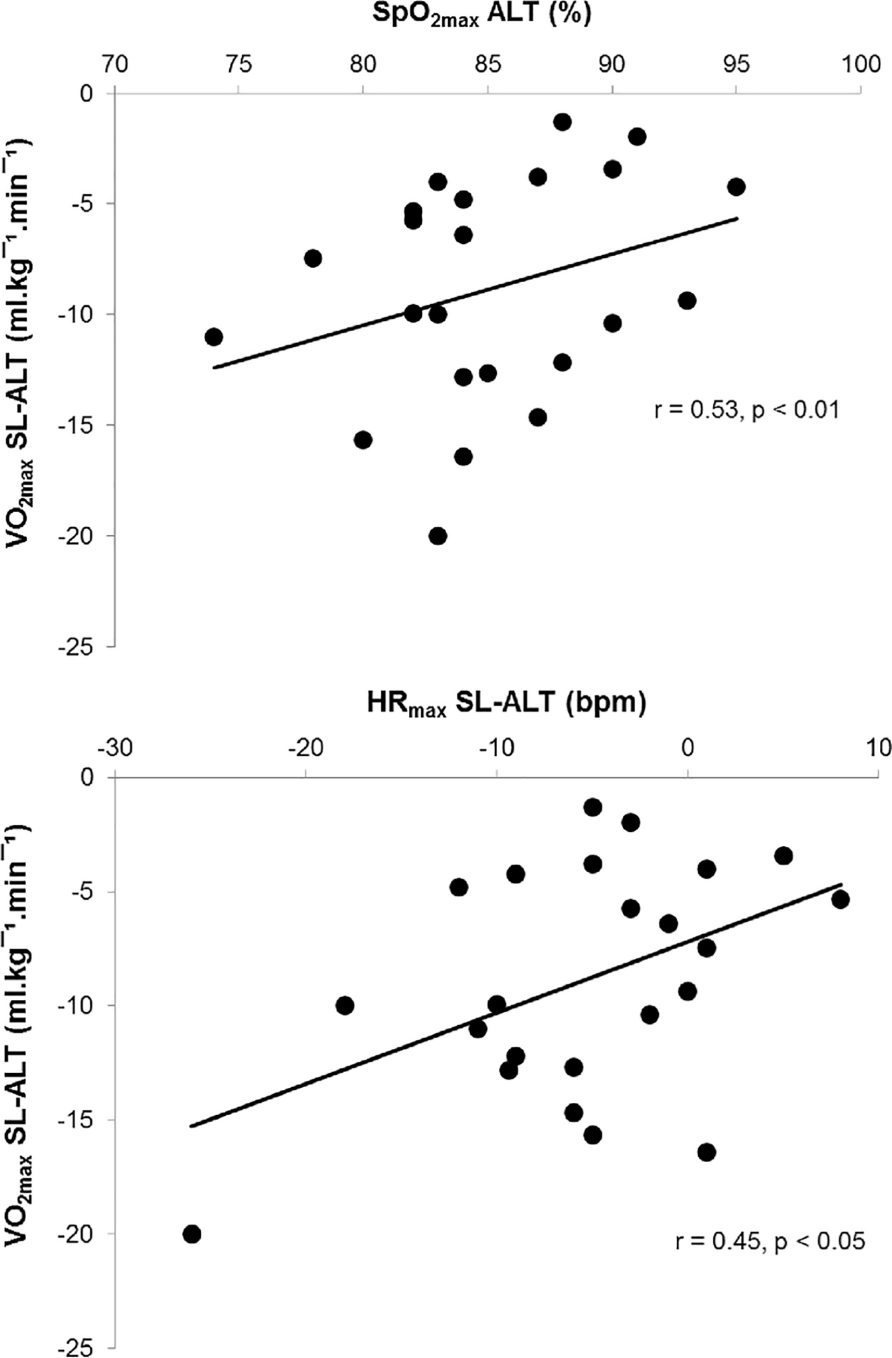
Correlations between decrease in VO_2max_ (VO_2max_ SL-ALT, difference between sea level and altitude values) with SpO_2_ values at the end of exercise at altitude (SpO_2max_ ALT) and with decrease in HR_max_ (HR_max_ SL-ALT, difference between sea level and altitude values). All participants were considered, n = 23.

VO_2max_, power achieved at VO_2max_ (P-VO_2max_) and duration of exercise at ALT were significantly lower than at SL in the three groups (Table 2). VO_2max_ SL-ALT (difference between SL and ALT values) was significantly greater in EIH group compared to non-EIH and untrained groups in relative values (-22 ± 7.9%, -16 ± 5.3% and -13 ± 9.4%, respectively) and in absolute values. P-VO_2max_ fall was around 10% in the three groups. Only EIH group exhibited a significant decrease of HR_max_ at ALT compared to SL condition (Table 2). When the data from all groups were pooled, there were significant relationship between VO_2max_ SL-ALT and SpO_2max_ at ALT (r = 0.53, p < 0.01) and also with HR_max_ SL-ALT (r = 0.45, p < 0.05; Fig 3). No significant effect of ALT has been observed for VE_max_, B_Fmax_ and V_Tmax_. B_Fmax_ and VE/VCO_2max_ were lower in EIH group compared to non-EIH and untrained groups. VE_max_ tended to be lower in EIH group compared to non-EIH group (p = 0.09).

## Discussion

The main finding of this investigation is that non-EIH group had the same O_2_ desaturation level at ALT with EIH athletes identified at SL. However EIH athletes have a higher decline in VO_2max_, HR_max_ and an exercise ventilatory response possibly attenuated.

### Methodological considerations

For ethical reasons this study used pulse oximetry, a non-invasive method to estimate SaO_2_ rather than blood arterial gases. Pulse oximetry method has been reported to deliver high precision, reproducibility and validity for O_2_ saturation above 75% when compared to O_2_ saturation measured from arterial blood gases at rest and exercise, at SL and at ALT [16,17]. Mollard et al. [17] have shown that the correlation between SaO_2_ and SpO_2_ was strong (r^2^ = 0.97, p < 0.001). In our study we are in agreement with the literature because SpO_2_ values were > 75%. Further we were very rigorous in the use of this method. Being aware of the limitations caused by the use of pulse oximetry, we used an EIH definition suitable at SpO_2_ measurement. Prefaut et al. [9] proposed that “EIH may be considered to exist when SaO_2_ decreases of 4% from baseline with 1 to 2% accounting for the accuracy of pulse oximeters and 1 to 2% accounting for the shift to the right of the oxyhemoglobin dissociation curve during exercise due to acidosis. EIH should persist for at least the last 3 steps of an incremental exercise test or the last 3 minutes of a steady state exercise test”.

### O_2_ desaturation in EIH athletes at ALT

At ALT, the decline in PIO_2_ leads to a decline in PO_2_ down the cascade from the atmosphere, to the alveoli, to the arterial blood and finally into the capillary. However, impairment in PaO_2_ during exercise at ALT may not be universal for all subjects. In fact previous studies have shown a greater arterial O_2_ desaturation in athletes at maximal exercise in hypoxia [5,18]. Chapman [12] suggested that when PaO_2_ corresponds to the shoulder of the oxyhemoglobin dissociation curve (as for EIH) a small decline in PO_2_ leads to a relatively large decline in O_2_ saturation. As SpO_2_ during maximal exercise at SL in EIH group was on the shoulder of the oxyhemoglobin dissociation curve, we hypothesized that in response to a same exercise done at moderate altitude can result a significantly greater SpO_2_ fall in EIH group. Yet in our study, the same ΔSpO_2_ has been observed at ALT between EIH and non-EIH athletes. Previous studies at higher altitude (3000–4000m) have reported controversial results but seem indicate a higher decline in SpO_2_ for EIH athletes compared to non-EIH athletes [4,14,15]. Our results suggest that at ALT (~ 2000 m), reduce in PIO_2_ is not sufficient to induce a greater O_2_ desaturation in EIH athletes compared to non-EIH athletes. Our study is the first at moderate altitude, thus more studies are necessary to make a general trend.

### Specific cardio-ventilatory responses of EIH athletes at ALT

At ALT, our results suggest an attenuate ventilatory response in EIH athletes but the small number of subjects in each group not allows us to clearly affirm this idea. In contrast, Gavin et al. [4] have clearly reported lower VE_max_, VE/VO_2max_ et VE/VCO_2max_ at 4000 m in EIH athletes. It has been generally accepted that the ventilatory response to hypoxia is mainly mediated by the sensitivity of peripheral chemoreceptors [19]. Thus, the attenuated pulmonary ventilation at ALT in EIH group could be related to an alteration of the ventilatory response to hypoxia (HVR), as an index of peripheral chemosensitivity to hypoxia. Indeed, previous studies [20,21] have indicated that EIH athletes demonstrate a lower HVR suggesting that inadequate hyperventilatory response at ALT must be due to low chemoresponsiveness. However these studies have not measured ventilatory parameters during exercise in hypoxia. A potential low chemoresponsiveness in EIH group during exercise at ALT can be a surprising result because intermittent hypoxia exposure (as during training in EIH group) has been repeatedly shown to increase HVR [22–25]. This discrepancy can be explained by at least two reasons. Firstly, the increase in HVR seems to enhance exercise ventilation and improve SaO_2_ depending on the altitude where exercise is performed, since it was observed at high altitude (4500 m) [22] but not at moderate altitude (2500 m or below) [26,27]. An explanation can be the level of arterial oxygenation. In fact mean SpO_2_ values of our subjects during exercise at moderate ALT were around 80% whereas at 4500 m the values were around 60% [22]. As a consequence the stimulus to the peripheral oxygen sensing chemoreceptors was markedly lower in our subjects and could have contributed only slightly to the regulation of exercise ventilation. Secondly, in general, athletes are known to express low exercise ventilation and are reported to illustrate low chemoresponsiveness at SL [28]. The blunted chemosensitivity to HVR in the endurance athletes has been considered to be due to endurance training over long periods [29]. Thus exercise training and exposure to intermittent hypoxia might have opposite effects on HVR. At 4500 m, Katayama et al. [30] have observed that HVR increased significantly in control group but not in training group. These results would suggest that endurance training during intermittent exposure to hypoxia depresses the increment of chemosensitivity to hypoxia. Thus, the larger relative hypoventilation in EIH group during exercise in moderate ALT could be due to a larger effect of aerobic training on chemoreceptor desensitization.

Concerning cardiac response, Mollard et al.[7] have noted that HR_max_ decreased from 1000 m in trained subjects and from 2500 m in untrained subjects. Our results confirm the decrease in HR_max_ at acute moderate ALT in EIH athletes but not in non-EIH athletes. This greater decrease in HR_max_ at ALT in EIH athletes compared to non-EIH athletes has been observed in previous studies but not at moderate ALT [14,15]. Autonomic changes could be involved in the reduction in HR_max_ at ALT in EIH group. Indeed, prior studies have provided a gradual down regulation of β-adrenergic receptors [31] and an up-regulation of muscarinic receptors [32] over the time of exposure to ALT, as a possible mechanism of myocardial adaptation to hypoxia. Further it has been shown that chronic exercise, as chronic exposure to hypoxia, reduced the number of cardiac β receptors [33]. Perhaps the frequent repetition of long-duration exercises with training in subjects who exhibit an arterial O_2_ desaturation could induce a similar mechanism of myocardial adaptation and could explain the decrease in HR_max_ at ALT in EIH group.

### Consequences of EIH on VO_2max_ at moderate altitude

At SL, EIH is already known to negatively affect VO_2max_ [8] and subsequently exercise performance [10]. In this study at 2150 m, EIH athletes demonstrated a higher decline in VO_2max_ from SL than non-EIH athletes. From an investigation compiling the results of 11 studies examining the magnitude of VO_2max_ decline with acute ALT exposure in endurance-trained athletes, VO_2max_ declines by 7.7% for every 1000 m ascended above SL [1]. The mean decrease in VO_2max_ in non-EIH athletes in this present study was 7.4% per 1000 m, which is in accordance with the literature. In EIH athletes, VO_2max_ fall is higher (10% per 1000 m) exceeding the standards reported in the literature. Recently, Chapman et al. [12] suggested that the degree of arterial desaturation during maximal exercise at SL, and not baseline VO_2max_ levels *per se*, is a primary limiting factor determining VO_2max_ decline with exposure to acute moderate ALT. But as previously described, SpO_2_ fall at moderate ALT was equal in both EIH and non-EIH groups, bringing into question the contribution of other limiting factor(s) determining VO_2max_ decline in EIH athletes. According to Fick equation, a fall in HR_max_ at ALT can have an effect on VO_2max_ via a reduction of maximal cardiac output (Q_max_). HR_max_ decrease under ALT was higher in EIH than in non-EIH athletes and even more compared to untrained participants. Thus greater VO_2max_ decrement in EIH athletes could be explained by a larger reduction in O_2_ transport than non-EIH athletes. However in the present study, Q_max_ had not been measured; the effects of modification of HR_max_ on Q_max_ should be interpreted with caution. Further although EIH group shows a significant decrease in HR_max_, this value is not significantly different between the two athletes groups at altitude.

This study has also shown a decrease of P-VO_2max_ at altitude around 10% despite a VO_2max_ decrease around 22% in EIH group. Thus it seems that the decrease of P-VO_2max_ at altitude is not proportional with the VO_2max_ decrease. As in our study, Peltonen and colleagues noted that hypoxia of 2 500 m reduced VO_2max_ nearly twice as much as maximal power during a maximal exercise on cycle ergometer [34]. Several studies have shown that chronic [35] and acute [36,37] lack of oxygen supply to muscle, reduces the leftwards shift of the electromyogram power spectrum density during sustained static contractions of 60–80% of maximum voluntary contraction. This reduction in the power spectrum density corresponds to a preferential recruitment of fast motor units [38], which may constitute an adaptive process to limit the recruitment of slow motor units which are highly oxygen dependent [36]. The preferential recruitment of fast motor units could constitute an adaptive muscle response to a reduced oxygen supply and can explain the lesser decrease of P-VO_2max_ compared to VO_2max_ decrease in hypoxia. Further investigations are needed to confirm this hypothesis.

## Conclusion

EIH athletes have shown a greater aerobic impairment than non-EIH athletes at altitude in spite of a same O_2_ desaturation level. Furthermore they also demonstrated a greater decrease in HR_max_ and potentially ventilatory adaptations at altitude. Thus, EIH athletes develop specific cardiorespiratory adaptations during exercise at acute moderate altitude maybe due to their frequent exposures to hypoxia. Based on these results, this study highlights the importance to discriminate EIH athletes in studies involving exercise adaptations in endurance athletes at altitude. Definitely further investigations into the mechanisms of EIH at moderate altitude are warranted.

## Supporting Information

S1 Fig Kinetics and delta of haemoglobin O_2_ saturation at sea level and at altitude for all the subjects. (XLSX)

S1 Table Anthropometric and training data for all the subjects. (XLSX)

S2 Table Incremental maximal exercise parameters at sea level and at altitude for all the subjects. (XLSX)
